# “WITH AGE COMES WISDOM:” A QUALITATIVE REVIEW OF ELDER PERSPECTIVES ON HEALTHY AGING IN THE CIRCUMPOLAR NORTH

**DOI:** 10.1093/geroni/igz038.3089

**Published:** 2019-11-08

**Authors:** Britteny M Howell, Jennifer R Peterson

**Affiliations:** University of Alaska Anchorage, Anchorage, Alaska, United States

## Abstract

Cross-cultural research has shown marked variation in health outcomes across the world’s older adult populations. Indeed, older adults in the Circumpolar North experience a variety of health disparities. Because aging is a biological process rooted in sociocultural context, there exists great variation in the ways older adults define and experience healthy, or “successful,” aging in their communities. The aim of this analysis was to synthesize qualitative research among older residents (aged 50+ years) in the Circumpolar North to identify a definition of healthy aging common in the region. A thorough review was conducted across a variety of academic search databases for peer-reviewed, qualitative studies conducted among community-dwelling older adults. The search strategy initially identified 194 articles; 23 articles met the inclusion criteria. Included studies were coded and analyzed using Grounded Theory to examine underlying themes of healthy aging in the Circumpolar North. The findings reveal the importance older adults place on respect, their relationship to the land, and psychosocial resilience into multidimensional models of healthy aging. We present a complex concept map demonstrating how healthy aging perspectives fit together into a multidimensional model of health in the Circumpolar North. This research also highlights the need for increased translational research with populations in the Circumpolar North that are under-represented in the literature.

